# Hypothyroidism may exacerbate valproate-related hyperammonemic delirium

**DOI:** 10.7603/s40681-016-0012-6

**Published:** 2016-05-09

**Authors:** Chung-Chieh Hung, Chieh-Hsin Lin, Hsien-Yuan Lane

**Affiliations:** 1 Graduate Institute of Clinical Medical Science, China Medical University, 404 Taichung, Taiwan; 2 Department of Psychiatry, China Medical University Hospital, 404 Taichung, Taiwan; 3 Department of Psychiatry, Chang Gung Memorial Hospital, 833 Kaohsiung, Taiwan

## 1. Introduction

Although it is well known that valproic acid (VPA) can induce hyperammonemia [1], few reports have been concerned with its relationship to hypothyroidism. Here we present a bipolar disorder patient with hypothyroidism (in fact, merely subclinical hypothyroidism) who experienced hyperammonemic delirium during VPA treatment.

## 2. Case

A 41-year-old woman was diagnosed with bipolar disorder at 31. After a thyroidectomy at 33 years old, she was found to have subclinical hypothyroidism: the thyroid-stimulating hormone (TSH) was 6.196 uIU/ml (reference range: 0.34-5.60) and free thyroxine (FT4) was 0.46 ng/dl (reference range: 0.54-1.40). No intervention was given due to her refusal. She started to participate in the day-care unit at 36 years old and received 1500mg/day of sodium valproate (1000 mg at 9 AM and 500mg at noon). Her VPA serum levels at 9 AM before taking her medication were between 44.3-54.8 ug/ml (reference range: 50-100), and her ammonia was at 60 ug/dl (reference range: < 70). With the medication she had both stable physical and psychiatric conditions.

One day, when she was 37, she was going about her routine life without any clinical symptoms after taking the 500mg of VPA at noon. Then, she suddenly experienced deteriorated consciousness at 2 PM and, consequently, delirium at 4 PM. Her ammonia soared to 700 ug/dl, and her VPA level was 130.3 ug/ml. TSH concentration increased to 8.867 uIU/ml, while FT4 remained similar (0.47 ng/dl). Other lab data showed negative findings. She remained delirious, her ammonia decreased to 352 ug/dl at 6PM with supportive care at the Emergency Department. She was hospitalized. Valproate was discontinued. The next morning, her consciousness improved partially, ammonia declined to 86 ug/ dl and VPA fell to 73.9 ug/ml at 9AM.

400 mg/day of Carbamazepine was given to the patient to treat her bipolar disorder, reaching a level of 6.8 ug/ml in the patient’s blood after one week. Meanwhile, her ammonia descended to 45 ug/dl. She was discharged one month later. With thyroxine supplement during the outpatient follow-up, her FT4 and TSH returned to a normal range after three months. Her ammonia further dropped to 12 ug/dl after one year of thyroxine supplement. No recurrent hyperammonemia was found during the following two years.

## 3. Discussion

An earlier case report described aggravation of hypothyroidism after carbamazepine and valproate treatment in a woman with hypothyroidism who was taking a levothyroxine supplement [2]. The mechanism may be competition between VPA and thyroid hormones [3]. However, valproate treatment didn’t alter thyroid hormones in another study [4]. In the patient considered in this report, TSH concentration increased marginally, while FT4 kept constant. More studies are suggested.

There was a report of an 82-year-old woman with hypothyroidism suffering from a hyperammonemic coma related to the discontinuation of levothyroxine therapy [5]. Another case report showed that thyroid hormones relieved hyperammonemia in a 53- year-old woman with hypothyroidism [6]. In our patient, VPA concentration dropped initially concomitant to the removal of valproate and then through thyroxine supplement.

The exact mechanism of hypothyroidism aggravating VPAinduced hyperammonemia remains unknown. Thyroid hormones are hypothesized to regulate hepatic mitochondrial catabolism [7]. As of now, we should be more cautious when prescribing valproate to patients with hypothyroidism, even if it is merely subclinical hypothyroidism.

## References

[1] Lewis C, Deshpande A, Tesar GE, Dale R. (2012). Valproate-induced hyperammonemic encephalopathy: a brief review. Curr Med Res Opin.

[2] Krysiak R, Stojko R. (2014). Transient hypothyroidism induced by anticonvulsant agents. Neuro Endocrinol Lett.

[3] Benedetti MS, Whomsley R, Baltes E, Tonner F. (2005). Alteration of thyroid hormone homeostasis by antiepileptic drugs in humans: involvement of glucuronosyltransferase induction. Eur J Clin Pharmacol.

[4] Isojarvi JI, Turkka J, Pakarinen AJ, Kotila M, Rattya J, Myllyla VV (2001). Thyroid function in men taking carbamazepine, oxcarbazepine, or valproate for epilepsy. Epilepsia.

[5] Rimar D, Kruzel-Davila E, Dori G, Baron E, Bitterman H. (2007). Hyperammonemic coma–barking up the wrong tree. J Gen Intern Med.

[6] Hitoshi S, Terao Y, Sakuta M. (1993). Portal-systemic encephalopathy and hypothalamic hypothyroidism: effect of thyroid hormone on ammonia metabolism. Intern Med.

[7] Tajiri J, Shimada T, Naomi S, Umeda T, Sato T. (1984). Hepatic dysfunction in primary hypothyroidism. Endocrinol Jpn.

